# Early swelling response to phytohemagglutinin is lower in older toads

**DOI:** 10.7717/peerj.6104

**Published:** 2018-12-20

**Authors:** Francisco Javier Zamora-Camacho, Mar Comas

**Affiliations:** 1 Department of Biological Sciences, Dartmouth College, Hanover, NH, United States of America; 2 Department of Biogeography and Global Change, Museo Nacional de Ciencias Naturales (MNCN), Spanish National Research Council (CSIC), Madrid, Spain; 3 Department of Integrative Ecology, Estación Biológica de Doñana (EBD), Spanish National Research Council (CSIC), Sevilla, Spain

**Keywords:** Ageing, Amphibian, *Epidalea calamita*, Global decline, Phytohaemagglutinin

## Abstract

The effects of age on performance of life-history traits are diverse, but a common outcome is senescence, an irreversible deterioration of physical and physiological capabilities of older individuals. Immune response is potentially bound to senescence. However, little is known about immune response ageing in amphibians. In this work, we test the hypothesis that amphibian early immune response is reduced in older individuals. To this end, we captured adult natterjack toads (*Epidalea calamita*) and inoculated them with phytohemagglutinin, an innocuous protein that triggers a skin-swelling immune response whose magnitude is directly proportional to the ability of the individual to mount an immune response. We measured early swelling immune response (corresponding to an innate-response stage) hourly, for six hours, and we calculated the area under the curve (AUC) for each individual’s time series, as a measure of immune response magnitude incorporating time. We estimated toad age by means of phalanx skeletochronology. Swelling and AUC decreased with age. Therefore, in accordance with our predictions, early immune response seems subject to senescence in these toads. Reduced ability to get over infections due to senescence of immune respose might be—together with a worse functioning of other organs and systems—among the causes of lower survival of older specimens.

## Introduction

Performance of life-history traits shifts with age in most organisms (Stearns, 1992), as physiological and ethological processes change throughout organisms’ lifetime (Kirkwood & Austad, 2000). However, ageing may affect differently various life-history traits in different organisms (Jones et al., 2014), enhancing some while impairing others in the same individual, implying different genetic and physiological trade-offs (Massot et al., 2011; González-Tokman et al., 2013). Also, the effect of ageing on a given trait may depend on the ontogenetic stage of organisms (Partridge & Gems, 2006). Although processes underlying ageing are rather intricate, some common patterns can be recognized. Ageing typically results in senescence, an irreparable decay of physical and physiological conditions (Nussey et al., 2013), which compromises performance (MacNulty et al., 2009), health (Møller & De Lope, 1999), and survivorship (Monaghan et al., 2008) in late life stages (Forslund & Part, 1995).

Although the potential genetic basis of senescence remains unclear (Charmantier et al., 2006; Brommer, Wilson & Gustafsson, 2007; Wilson, Charmantier & Hadfield, 2008), some agents involved have been identified (López-Otín et al., 2013). For instance, deleterious mutations accumulate with age (Kirkwood & Austad, 2000). Also, cumulative oxidative stress promotes senescence (Finkel & Holbrook, 2000). Oxidative stress is defined as the imbalance between pro-oxidant metabolites and anti-oxidant defences, with negative effects on animal health and physiological homeostasis (Halliwell, 2007). Indeed, oxidative stress decreases longevity (Costantini, 2014), and may play a role in reduced sexual attractiveness of older individuals (Torres & Velando, 2007). More generally, senescence could be a consequence of physical deterioration, which has proven to impair performance (Combes, Crall & Mukherjee, 2010). Environmental conditions may also affect: nutrient deprivation during early life has proven to accentuate senescence (Lee, Monaghan & Metcalfe, 2016). As senescence happens in late stages of individual lives, many individuals die before senescence, and/or have already reproduced when it happens, so the effects of selection on late life stages are mild (Rose, 1984).

Moreover, senescence often impairs reproductive output (e.g., Møller & De Lope, 1999; Moya-Laraño, 2002; Reed et al., 2008). However, according to the terminal investment hypothesis, reproductive effort might also be greater in older individuals, with expectancy of fewer future reproduction events (Williams, 1966; Clutton-Brock, 1984). Terminal investment hypothesis predicts that older individuals, with lesser residual reproductive value, will attempt optimized fitness by increasing current reproductive effort to the detriment of future reproduction events, as well as other energy-consuming life-history traits, since their probabilities of future reproduction events are low (Pärt, Gustafsson & Moreno, 1992).

Among traits susceptible to ageing, immune response is of particular interest, for several reasons (review in DeVeale, Brummel & Seroude, 2004). Despite its role on soma preservation and survival (Møller & Saino, 2004) by combating pathogens and parasites (Wakelin & Apanius, 1997), immune response entails a number of energetic and physiological costs (Schmid-Hempel, 2011). As a consequence, immune response may diminish reproductive success, growth, and even survival (Uller, Isaksson & Olsson, 2006; Bascuñán-García, Lara & Córdoba-Aguilar, 2010). Some of those costs might therefore be related to ageing. Firstly, immune activity may provoke oxidative damage (Lambeth, 2007; Sorci & Faivre, 2009), which could contribute to soma senescence (reviewed in Selman et al., 2012), including immune system itself (De la Fuente, 2002). Also, immune system activation increases energy expenditure (Martin, Scheuerlein & Wikelski, 2003). Indeed, energy-consuming traits are particularly likely to be affected by senescence, as mitochondrial respiration becomes dysfunctional in aged individuals, limiting energy metabolism (Navarro & Boveris, 2007). However, the relationship between age and immune response might be particularly intricate: for example, whilst immune system senescence could impair animal ability to overcome infections and parasites, at the same time older individuals could acquire immune memory, which may reduce infection rates in older animals (Raffel et al., 2009).

Overall, senescence occurs in most organisms. However, its rates vary considerably within and among species (Nussey et al., 2013), but this variation is poorly understood (Jones et al., 2014; Hammers et al., 2015). In animals in general, and in amphibians in particular, little is known about the trade-offs involving ageing processes affecting the immune system (Kara, 1994), although some components of it are known to decline with age (review in Torroba & Zapata, 2003). In this work, we test the hypothesis that older natterjack toads (*Epidalea calamita*) should elicit an early immune response (corresponding to an innate-response stage; Brown, Shilton & Shine, 2011) less intense than younger conspecifics. Moreover, we compare toads from natural habitats with conspecifics from agrosystems, since the latter show delayed early swelling response to PHA (Zamora-Camacho, in press), most likely due to the physiological stress caused by agrosystem conditions (Zamora-Camacho & Comas, 2017). Plus, we compare males and females, because the pattern of early swelling response to PHA is different between sexes in this species (Zamora-Camacho, in press), probably due to the greater reproductive investment of females through egg production (Tejedo, 1992), and/or the immunosuppresor effect of testosterone in males (Folstad & Karter, 1992).

## Materials and Methods

### Study species

*Epidalea calamita* is a medium-sized (54–75 mm snout-vent length -SVL- in this system) Bufonid toad that inhabits diverse habitats throughout Central and Southwestern Europe (Gómez-Mestre, 2014). Activity takes place mainly during warm, wet nights, and thus phenology varies according to climate: this species hibernates in habitats where winters are cold, but resorts to aestivation instead to avoid summer drought in southern warm locations (Gómez-Mestre, 2014). Similarly, there is variation in reproduction period, which may take place in late winter or in spring (Gómez-Mestre, 2014). Eggs are often laid in very small temporary ponds, and tadpoles can finish metamorphosis in periods as short as 6 weeks (Gómez-Mestre, 2014).

### Study area

Toads were captured in natural pine grove Pinares de Cartaya, and agroecosystems nearby, in the Southwest of Spain (37°20′N, 7°09′W). Pine grove was composed of stone pine (*Pinus pinea*) as the tree stratum, and *Pistacia lentiscus, Cistus ladanifer*, and *Rosmarinus officinalis* as an undergrowth. Although some debate exists on the autochthonous or introduced origin of stone pine in this area, their dominance in this ecosystem has been estimated for at least 4,000 years (Martínez & Montero, 2004). Agroecosystems were around 5 km away, in a traditional crop area, which in the recent years has shifted to intensive orange and strawberry plantations, among other crops. Small ponds where *E. calamita* reproduces were numerous throughout both habitats. Warm winter and arid summer climate in the area makes toads skip hibernation, but they undergo an inactivity period during the summer instead.

### Toad collection and management

We collected 18 female and 20 male *B. calamita* by hand during their mating season (January–April) in 2015. Toads were caught while active on rainy nights, or actively searched for under rocks or logs during the day. We distinguished males because their throats are purple-to-pink due to their vocal sacs, and because they show brown or black nuptial pads in their fingers and forelimb tubercles (Gómez-Mestre, 2014). For each toad, we measured SVL with a ruler to the nearest mm, body mass with a balance (model CDS-100, precision 0.01 g), and left forelimb sole pad thickness to the nearest 0.01 mm, using a pressure-sensitive micrometer (Mitutoyo). We took three consecutive measures in quick succession, and considered the mean as sole pad thickness (Brown, Shilton & Shine, 2011). Afterwards, we subcutaneously injected 0.1 mg of phytohemagglutinin (PHA, Sigma Aldrich L-8754; Sigma Aldrich, St. Louis, MO, USA) diluted in 0.01 ml phosphate buffer saline in left forelimb sole pad. We inoculated the same amount of PHA to all individuals because there were no significant differences in body mass between toads from both habitats (*F*_1,37_ = 0.146; *P* = 0.704). PHA is a protein that provokes a safe skin-swelling immune response that involves T-cells and other components of the immune system (Kennedy & Nager, 2006; Martin et al., 2006; Bílková, Vinklerová & Vinkler, 2015). The magnitude of that swelling is directly proportional to the capacity of the individual to elicit an immune response (Parmentier, De Vries Reilingh & Nieuwland, 1998; Vinkler, Bainova & Albrecht, 2010; Clulow, Harris & Mahony, 2015). This method has proved valid in amphibians (Brown, Shilton & Shine, 2011; Clulow, Harris & Mahony, 2015). According to Smits, Bortolotti & Tella (1999), omitting phosphate buffer saline (PBS) controls in PHA tests has little impact on the results, decreases handling errors, and reduces the coefficient of variation resulting from measurement inaccuracy. For this reason, PHA tests are often simplified by not using PBS controls in amphibians (Gervasi & Foufopoulos, 2008; Iglesias-Carrasco, Martín & Cabido, 2017; Zamora-Camacho, in press) as well as in other groups (v.gr. Martin, 2005; Hale & Briskie, 2007; Moreno-Rueda, 2010; Moreno-Rueda & Redondo, 2012).

After inoculations, we measured sole pad thickness as described, once per hour, for the six hours subsequent to inoculations. Early swelling response to PHA in this species peaks before six hours from inoculation (Zamora-Camacho, in press). This procedure allowed us to calculate swelling immune response on an hourly basis, by subtracting sole pad thickness prior to inoculations to sole pad thickness at each hourly measure after inoculation for each individual. At these early stages, swelling response to PHA is directly proportional to recruitment of macrophages, eosinophils and neutrophils, which corresponds to an innate response in closely-related *Rhinella marina* toads (Brown, Shilton & Shine, 2011). With that information, for each individual, we calculated area under the curve (AUC, in h*mm) resulting from swelling-response progression during the six hours measured, as a directly proportional measurement of swelling response magnitude incorporating time (Fekedulegn et al., 2007). We obtained AUC with software GraphPad Prism 7.0, which applies the trapezoidal formula. During the whole procedure, we kept toads in individual plastic terraria, with humid peat as a substrate and an opaque piece of plastic as a shelter. Toads were released at their capture sites after the last measure.

### Skeletochronology technique

Prior to their release, we clipped two toes from each toad, disinfecting the wounds with chlorhexidine and stanching them with a tissue adhesive glue (Dermabond). Toes were used for age estimates, as previously described in Zamora-Camacho & Comas (2017). Specifically, age was estimated by means of phalanx skeletochronology (Comas et al., 2016) using one phalanx per toad. This method does not involve killing specimens. Phalanx skeletochronology is based on the growth pattern of bones of indeterminate-growth ectotherms. Cross-sections of the bones appear surrounded by a line (lines of arrested growth, hereafter LAGs) delimiting areas of rapid osteogenesis when growth is slow or does not happen, typically during hibernation and/or aestivation. Age can be estimated by couting these LAGs: each LAG correspond to one period of inactivity. Since toads in this area only stop activity, and therefore growing, once a year during aestivation, each LAG corresponds to one year. We confirmed the number of LAGs corresponding to one year by calibrating the technique with six subadults whose age was known, which we did not use in the analyses.

We preserved phalanges in 70% ethanol. We performed several trials to estimate the time needed for decalcification. Based on those trials, we decalcified bones by immersing them in 3% nitric acid for 150 min. A solution of phosphate-buffered saline solution with sucrose was used to preserve decalcified samples for at least 48 h at 4 °C. Then, we sectioned them at 16 µm using a freezing microtome (HM500, MICROM) at the Estación Biológica de Doñana (EBD-CSIC), Seville (Spain). We stained cross-sections for 20 min with Harris hematoxylin. After that, we washed the slides in tap water for 5 min to rinse stain. Finally, we used an alcohol chain to dehydrate sections, which we mounted on slides and fixed with DPX (a common medium for mounting histological samples).

Afterwards, we counted the number of LAGs in each cross-section with a light microscope (Leitz Dialux20) at magnifications from 50 to 125X. To do so, we used several representative cross-sections among those in which LAGs were clearly visible, and photographed them several times with a ProgRes C3 camera. For better observation of LAGs, we only photographed bone diaphises. More specifically, we focused on sections were medullar cavity was at its minimun size, while that of the periosteal area was at its maximum size (Comas et al., 2016). Next, the same researcher counted the amount of LAGs in the periosteal bone, on two independent occasions; in no case did the researcher know the identification of the toad studied (Comas et al., 2016). Readings matched in all cases. Because we captured toads during winter and spring, LAGs formed during the preceding summer inactivity period could be discerned from the exterior border of the bones, and were not counted.

### Statistics

Since data met the criteria of residual homoscedasticity and normality, we conducted parametric statistics (Quinn & Keough, 2002). Firstly, we performed two simple regressions with AUC as a dependent variable, and age in the first case and SVL in the second case as independent variables. Then, we performed a multiple regression with AUC as a dependent variable, and SVL and age as independent variables. Finally, we conducted a series of ANCOVAs to test the effects of SVL and age on AUC and each hourly measurement independently with sex and habitat introduced as factors—since both affect swelling immune response of these toads (Zamora-Camacho, in press)—without non-significant habitat*sex interactions. Statistical analyses were performed with the software Statistica 8.0.

## Results

**Figure 1:**
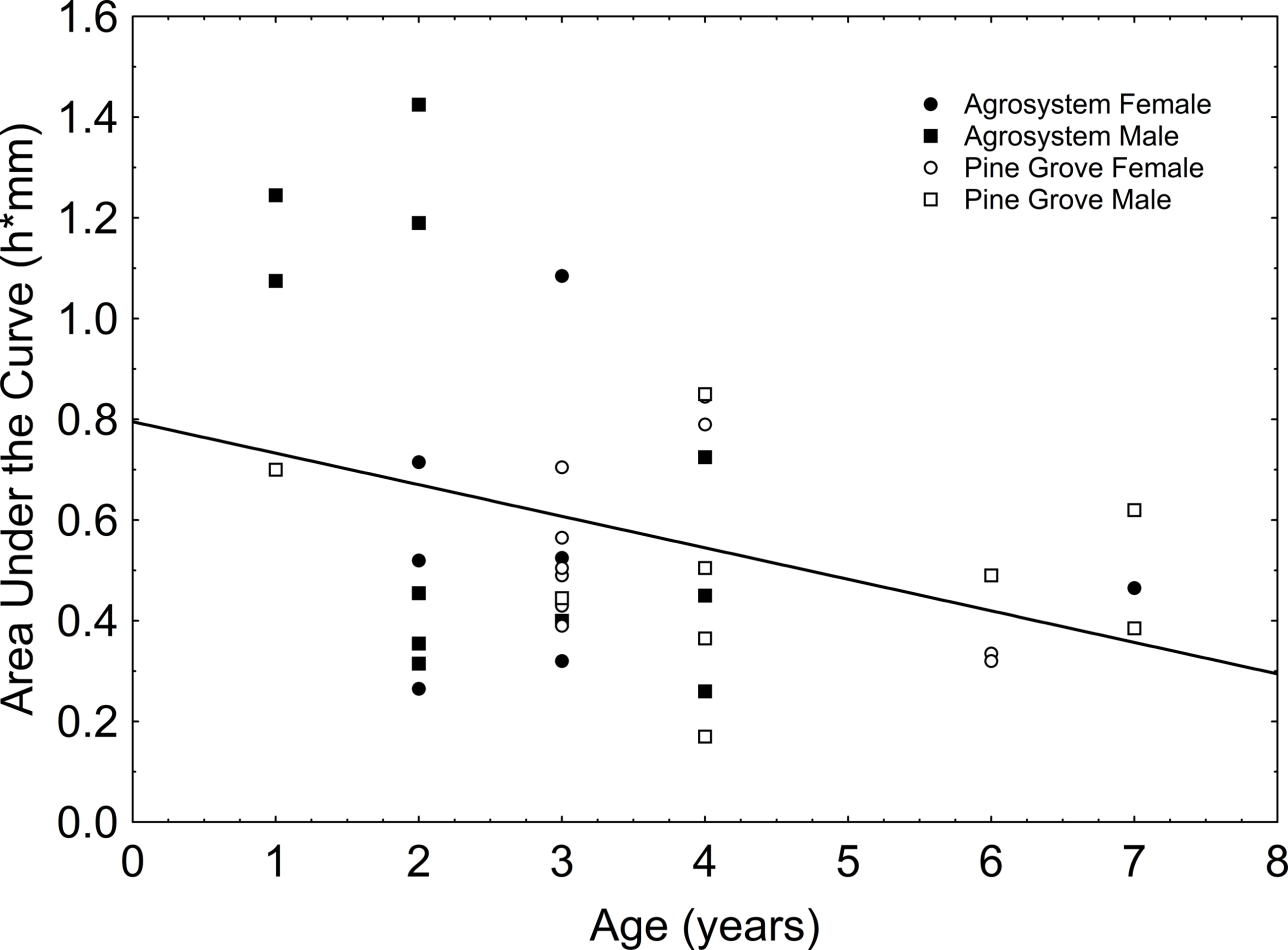
Relationship between age and area under the curve of *Epidalea calamita* toads. Black figures represent agrosystem toads, and empty figures represent pine grove toads. Circles represent females, and squares represent males.

AUC showed a significant negative correlation with age (*F*_1,36_ = 4.867; *β* =  − 0.345; *r*^2^ = 0.119; *P* = 0.034; Fig. 1). Relationship between the immune response and SVL was not significant (*F*_1,36_ = 0.184; *β* =  − 0.071; *r*^2^ = 0.005; *P* = 0.670). Moreover, when age and SVL were simultaneously analysed in a multiple regression on AUC (marginally non-significant: *F*_2,35_ = 3.203; *r*^2^ = 0.155; *P* = 0.053), we detected a significant relationship of AUC with age (*F*_1,35_ = 6.195; *β* =  − 0.498; *P* = 0.018), but not with SVL (*F*_1,35_ = 1.474; *β* = 0.243; *P* = 0.233). Lastly, we found no effect of habitat (*F*_1,33_ = 0.001; *P* = 0.979), sex (*F*_1,33_ = 0.533; *P* = 0.470), and SVL (*F*_1,33_ = 1.189; *P* = 0.283) on AUC, while the effect of age on AUC was significant (*F*_1,33_ = 4.219; *P* = 0.048). Swelling one and six hours after inoculations showed a significant negative relationship with age (Table 1).

**Table 1:** Models showing the effects of age on hourly sole pad swelling during six hours following inoculations. In all models, sex and habitat were controlled for. Sex*habitat interactions were non-significant and thus excluded in all cases. *F*-, *β*-, and *P*-values are shown. Significant results are in bold. Sample size was 38 toads.

**Hours from inoculation**	**Age**			**Sex**		**Habitat**	
	*F*_1,34_	*β*-value	*P*-value	*F*_1,34_	*P*-value	*F*_1,34_	*P*-value
1	**4.136**	**−0.359**	**0.049**	1.491	0.230	0.694	0.411
2	1.628	−0.237	0.211	0.252	0.619	0.241	0.627
3	2.448	−0.286	0.127	0.278	0.601	1.926	0.174
4	2.288	−0.226	0.140	**5.177**	**0.029**	**6.144**	**0.018**
5	1.920	−0.251	0.175	0.197	0.660	0.312	0.580
6	**9.500**	**−0.520**	**0.004**	0.151	0.700	1.764	0.193

## Discussion

Our results show lower early swelling response to PHA in older individuals. Matching with our predictions, these findings suggest that ageing might impair toad immune capacity, aligned with a senescence pattern, at least during the early stages of the response, which correspond to an innate response. Supporting senescence of immune system in toads, some studies have found deteriorated immune organs and reduced immune-cell counts in older *Bufotes viridis* toads (Saad et al., 1994).

Eliciting an immune response is indeed energetically costly (Demas et al., 2012), and such energy challenge can be harder to afford by older individuals, whose energy metabolism is impaired (Green, Galluzzi & Kroemer, 2011). However, a fit physiological state and favourable ecological circumstances may reverse that trend (Palacios et al., 2011). For instance, Massot et al. (2011) found that immune response to PHA was higher in older than in younger female common lizards, *Zootoca vivipara*. Thus, immune response might be optimised (Graham, Allen & Read, 2005) in older individuals, in which reducing innate response could be compensated with a bolstered adaptive response as a consequence of accumulated antigen experiences (Franceschi et al., 2007; Janeway et al., 1999). However, other studies have found negative effects of age also on adaptive response (Solana, Pawelec & Tarazona, 2006; Weng, 2006), so a general pattern remains unclear. On the other hand, older individuals could have difficulties in facing oxidative stress caused by immune response (Constantini & Møller, 2009), so a milder immune response could reduce oxidative unbalance. In fact, oxidative stress induces senescence and favours age-related disorders (Hosokawa, 2002).

An alternative, but not mutually exclusive explanation implies a trade-off between immune capacity and reproduction. Limitations in energy-metabolism outcome could be on the basis of reduced immune response in older individuals (Hipkiss, 2008). According to the terminal investment hypothesis, older individuals could increase energy investment in current reproduction, since their chances of future reproduction events are low (Poizat, Rosecchi & Crivelli, 1999). In that context, the energy-limiting situation due to deteriorated metabolism in older individuals (Balaban, Nemoto & Finkel, 2005) could lead to trade-offs favouring reproduction to the detriment of other energy-consuming life-history traits (González-Tokman, González-Santoyo & Córdoba-Aguilar, 2013), such as immune response. Accordingly, immune-system activity has proven reduced as a consequence of terminal investment resource reallocation into reproduction (Krams et al., 2011). Conversely, an immune challenge can trigger terminal investment (Bonneaud et al., 2004; Nielsen & Hokman, 2012), suggesting complex relationships between immune system and reproduction in the context of ageing. Indeed, reproduction and immune activity are under a trade-off in great tit (*Parus major*) females (Ots & Horak, 1996), as could also be happening in this system, where some indicators of reproductive investment are higher in older individuals in *E. calamita* toads (Zamora-Camacho & Comas, 2017).

Interestingly, the negative effect of age on immune response affected similarly males and females, so we found no evidence of a sex-dependent mechanism underlying immune response senescence. Although we detected no sex differences in the relationship between immune response and age, they were expectable. On the one hand, the investment of female anurans in reproduction is particularly high (Yu et al., 2017), so the energy trade-off, likely more intense in older, energy-limited individuals (Navarro & Boveris, 2007; Green, Galluzzi & Kroemer, 2011), could be greater in females. Nonetheless, in agreement with our results, reproductive investment of *Hyla intermedia* female frogs shows no trade-off with age (Cadeddu & Castellano, 2012), and *Rana temporaria* female frogs do not trade off reproductive investment and growth (Lardner & Loman, 2003). On the other hand, the immunocompetence handicap hypothesis predicts lower immunocompetence in males due to the immunosuppressive effect of testosterone (Folstad & Karter, 1992). Nevertheless, we found no evidence for such handicap, nor a relationship with age. Accordingly, *Hyla arborea* male frogs showed no negative effect of testosterone injection on swelling response to PHA (Desprat et al., 2015). Indeed, immune responsiveness to PHA has often been found similar in male and female anurans (Zhang et al., 2017), also in this species (Zamora-Camacho & Comas, 2017).

Furthermore, we detected no effect of habitat on the negative relationship between age and early immune response. These agrosystem toads live shorter but grow larger and invest more in reproduction (Zamora-Camacho & Comas, 2017), and at the same time they show a delayed early swelling response to PHA in comparison with natural-habitat toads (Zamora-Camacho, in press). Therefore, we might expect accelerated reduction of early immune response in agrosystem toads due to agrosystem stressors. However, the tendency of early immune response with age was similar in both habitats.

In any case, reduced immune capacity in older individuals, at least in early stages according to our results, could be a consequence of senescence through mechanisms that remain obscure (reviewed in Torroba & Zapata, 2003), and at the same time play a role on increased mortality in older individuals (Hillyer et al., 2005). Older toads in this system showed reduced early immune response to PHA, which might suggest impaired ability to overcome infections (Hawley & Altizer, 2011) and parasites (Campião, Da Silva & Ferreira, 2009), as detected in other species. Therefore, deterioration of early swelling response, corresponding to innate immunity may be at least one of the causes of compromised survival in older adults (Zamora-Camacho & Comas, 2017), as immune response is directly related to survival (Møller & Saino, 2004). However, we cannot completely discard a cohort effect (potential effects caused by inter-annual differences in factors such as temperature, precipitation regimes, food availability, or pathogen exposure), as we measured age with skeletochronology—which implies a snapshot of the age structure at the time when animals were sampled—not with a longitudinal study. The experimental design described here is cross-sectional, since it explores characteristics of a population at a given moment (Nussey et al., 2008). On the other hand, longitudinal studies measure the same characteristics of the same individuals at different points of their lives, and are valuable for studying ageing processes, as they avoid biases by differential mortality (Nussey et al., 2008). However, we conducted a cross-sectional study instead because an immune challenge could have an effect on subsequent ones, which would reduce the reliability of repeated-measures results (Boughton, Joop & Armitage, 2011).

## Conclusions

In conclusion, early swelling response to PHA, corresponding to a preliminary innate response, is reduced in older individuals, which might suggest a senescence pattern. Along with deterioration of other organs and systems, senescence of immune response could play a role in reduced survival of older adults, by impairing their ability to overcome infections and parasites.

##  Supplemental Information

10.7717/peerj.6104/supp-1 Data S1 Raw data for analyses of senescence of immune response

## Additional Information and Declarations

### Field Study Permissions

The following information was supplied relating to field study approvals (i.e., approving body and any reference numbers): Junta de Andalucía granted permission for this study (AWG/MGD/MGM/CB).

### Data Availability

The following information was supplied regarding data availability: The raw data are provided in the Supplementary File.
